# Wrist-worn Accelerometry for Runners: Objective Quantification of Training Load

**DOI:** 10.1249/MSS.0000000000001704

**Published:** 2018-07-30

**Authors:** VICTORIA H. STILES, MATTHEW PEARCE, ISABEL S. MOORE, JOSS LANGFORD, ALEX V. ROWLANDS

**Affiliations:** 1 Sport and Health Sciences, College of Life and Environmental Sciences, University of Exeter, Exeter, UNITED KINGDOM;; 2 Cardiff School of Sport and Health Sciences, Cardiff Metropolitan University, Cardiff, UNITED KINGDOM;; 3 GENEActiv, Activinsights, Cambridgeshire, UNITED KINGDOM;; 4 Diabetes Research Centre, University of Leicester, Leicester, UNITED KINGDOM;; 5 National Institute for Health Research (NIHR), Leicester Biomedical Research Centre, Leicester, UNITED KINGDOM; and; 6 Alliance for Research in Exercise, Nutrition and Activity (ARENA), Sansom Institute for Health Research, Division of Health Sciences, University of South Australia, Adelaide, AUSTRALIA

**Keywords:** WORKLOAD, TRAINING EXPOSURE, TRAINING PROGRAMS, ATHLETE MONITORING, INJURY PREVENTION, PERFORMANCE

## Abstract

**Purpose:**

This study aimed to apply open-source analysis code to raw habitual physical activity data from wrist-worn monitors to: 1) objectively, unobtrusively, and accurately discriminate between “running” and “nonrunning” days; and 2) develop and compare simple accelerometer-derived metrics of external training load with existing self-report measures.

**Methods:**

Seven-day wrist-worn accelerometer (GENEActiv; Activinsights Ltd, Kimbolton, UK) data obtained from 35 experienced runners (age, 41.9 ± 11.4 yr; height, 1.72 ± 0.08 m; mass, 68.5 ± 9.7 kg; body mass index, 23.2 ± 2.2 kg·m^−2^; 19 [54%] women) every other week over 9 to 18 wk were date-matched with self-reported training log data. Receiver operating characteristic analyses were applied to accelerometer metrics (“Average Acceleration,” “Most Active-30mins,” “Mins≥400 m*g*”) to discriminate between “running” and “nonrunning” days and cross-validated (leave one out cross-validation). Variance explained in training log criterion metrics (miles, duration, training load) by accelerometer metrics (Mins≥400 m*g*, “workload (WL) 400-4000 m*g*”) was examined using linear regression with leave one out cross-validation.

**Results:**

Most Active-30mins and Mins≥400 m*g* had >94% accuracy for correctly classifying “running” and “nonrunning” days, with validation indicating robustness. Variance explained in miles, duration, and training load by Mins≥400 m*g* (67%–76%) and WL400–4000 m*g* (55%–69%) was high, with validation indicating robustness.

**Conclusions:**

Wrist-worn accelerometer metrics can be used to objectively, unobtrusively, and accurately identify running training days in runners, reducing the need for training logs or user input in future prospective research or commercial activity tracking. The high percentage of variance explained in existing self-reported measures of training load by simple, accelerometer-derived metrics of external training load supports the future use of accelerometry for prospective, preventative, and prescriptive monitoring purposes in runners.

Runners are suggested to be particularly at risk of developing a running-related injury (RRI) if they have one or a combination of the following: a history of injury, low or high running experience (high indicates that long distances have been run for many years), a low (women) or high (men) weekly training frequency, a low or high overall weekly running mileage or a sudden increase in training load (1–3). Characteristics of external training load (work done) typically described as the distance, frequency, intensity, and duration of running per day/week or month are therefore highly modifiable risk factors for RRI (1–4). Optimal patterns of training load relative to rest and sleep (recovery) are also important in the prevention of RRI and illness (5–7). However, a single validated method enabling longitudinal training patterns to be objectively, accurately, and unobtrusively quantified in runners is unavailable. A more detailed understanding of the influence of training load on RRI and performance could be enhanced by an improved ability to objectively monitor simple, yet meaningful characteristics of external training load in runners on a large population scale (5,8,9).

Within research and applied settings, characteristics of external training load, such as miles and duration, are typically recorded using a training log (self-reported or coach-reported), global navigational satellite system (GNSS), or prescribed within a training program. To avoid inaccuracies from self-reported data due to recall bias (overreported/underreported training/activity), characteristics of external training load can be more accurately quantified using objective measurements (9,10). For example, initial findings from the use of pedometers in military recruits to estimate distances covered over consecutive weeks of training have highlighted the importance of capturing evidence of previously unreported additional training and habitual physical activity (PA) associated with stress fractures (9). In addition to pedometers, there has been a vast increase in the use of more sophisticated, commercial, consumer-focused wrist-worn activity trackers that are worn 24/7 to monitor habitual PA (11). These usually incorporate accelerometers that sample at various frequencies and/or GNSS. With or without additional user input to improve the accuracy of identifying training events, the external training characteristics objectively recorded by the majority of these devices seem only to replicate those captured in a training log, for example, distance and duration. Restricting objective quantification of training characteristics to the replication of existing metrics may limit insight into the possible effects of accelerometer-derived metrics of external training load on performance and injury outcomes in runners.

Accelerometer-derived measures of load, if available, tend to input accelerations to “black-box” on-board processors and produce manufacturer-specific, proprietary metrics that appear difficult to interpret (5). For example, in team sports (12–15), there has been some development with the use of vest-/back-mounted triaxial accelerometers to provide a proprietary measure termed PlayerLoad™ (modified vector magnitude in arbitrary units representing rates of change in instantaneous acceleration (16)). Proprietary metrics limit comparisons with data recorded by other devices. The wear location and limited battery life in these devices also limits their ability to monitor other important training or nontraining activity outside of training sessions. These aspects, alongside other practical issues related to access to longitudinal data, limit the use of accelerometry in running-related research that seeks to develop new measures of external training load that might help reduce RRI and improve performance. The ability to objectively, unobtrusively, and accurately quantify external training load without user input using high-resolution, triaxial, open source (nonproprietary) acceleration data from a single wearable device over weeks at a time, is therefore attractive.

Wrist-worn accelerometers are now widely used in very large research cohorts to measure characteristics of habitual PA (17) including sleep without the need for a sleep diary (18). These research-grade monitors generate high-resolution raw data, which can be processed using open-source software, facilitating the development of metrics most appropriate for a specific research question. For example, outputs from these monitors have been validated with ground reaction force data (19,20) enabling metrics indicative of external mechanical loading relative to bone health to be established (21). A similar approach could therefore be developed to provide a field-based proxy measure of external mechanical load (biomechanical risk factor) relevant to injury. Example metrics in PA and health research include “Average Acceleration,” “Most Active-30mins” or “Mins≥400 m*g*” (Table 1) which describe the intensity of activity in different user-defined periods or time spent above user-defined intensities of activity (e.g. 400 m*g* is a validated vigorous activity threshold in adults (23)). Although these wrist-worn triaxial accelerometer-derived metrics are validated for use in large-scale population PA research, it is not yet known whether they can be used to accurately and unobtrusively measure external training load in runners in the field. The application of these sample metrics provides a justifiable starting point for objectively classifying and quantifying an alternative measure of external training load in runners. Further experimentation with the creation of a composite metric of workload (WL400–4000 m*g*; Table 1) from intensity multiplied by duration (25), may also provide a possible accelerometer-derived alternative to Foster’s (24) composite measure of training load (RPE × duration). Embedding a procedure for classifying running and nonrunning training days from accelerometer data and accurately obtaining accelerometer-derived metrics of external training load within existing, validated protocols for accurately monitoring habitual activity (26,27) including those used to derive accurate measures of sleep (18), would benefit subsequent analysis of patterns of training relative to rest and recovery (6,7). The benefits of high wear compliance and increased measurement reliability associated with the use of wrist-worn monitors (22) would also support this future analysis.

**TABLE 1:**
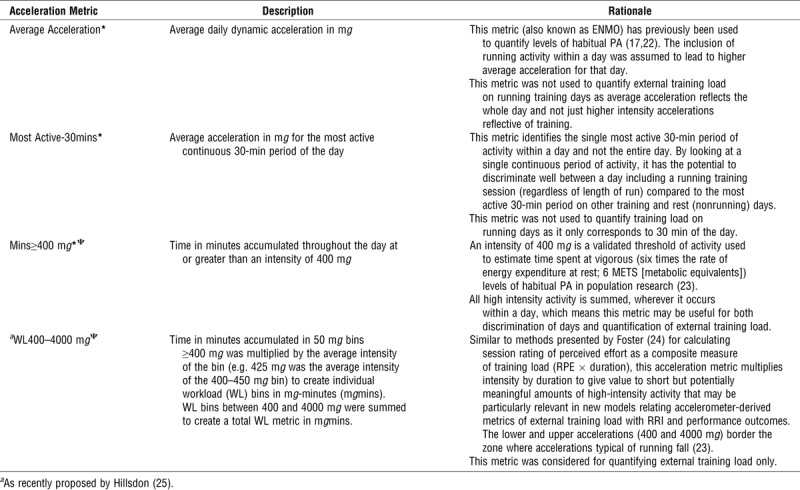
Acceleration metrics considered* for discriminating between running and nonrunning days and used^Ψ^ to quantify external training load on running training days.

The aims of this study are to assess whether simple PA metrics derived from the application of open-source analysis code to repeated week-long raw habitual PA data from wrist-worn tri-axial accelerometers in runners can be used to 1) objectively, unobtrusively and accurately discriminate between running training days and nonrunning days; and 2) quantify external training load on running training days. It was hypothesized that the Most Active 30mins metric (Table 1) would be the best discriminator for classifying running and nonrunning days as it focuses on a single continuous period of activity rather than an average derived from the entire day. It was also hypothesized that Mins≥400 m*g* and WL400–4000 m*g* would demonstrate at least a moderate level of correspondence (variance explained) with existing self-reported measures of training load (criterion measures) from a training log.

## METHODS

### Participants

Forty-one runners (22 women) with >2 yr running experience who were training for an event (e.g. 10 km, half/full marathon) were recruited. An early attrition rate (14.6%) due to injury or withdrawal resulted in 35 runners (19 women [54%]; Table 2) monitoring their training load for at least nine consecutive weeks (mean 12.6 ± 2.3 wk) between December 2015 and June 2016, to obtain a range of training intensities before their target event. Variations in average weekly running mileage, duration and pace (minutes per mile) averaged over the monitoring period indicate heterogeneous characteristics of training in this sample of runners (Table 2). The Sport and Health Sciences Ethics Committee at the University of Exeter approved this study, and all participants provided written informed consent.

**TABLE 2:**
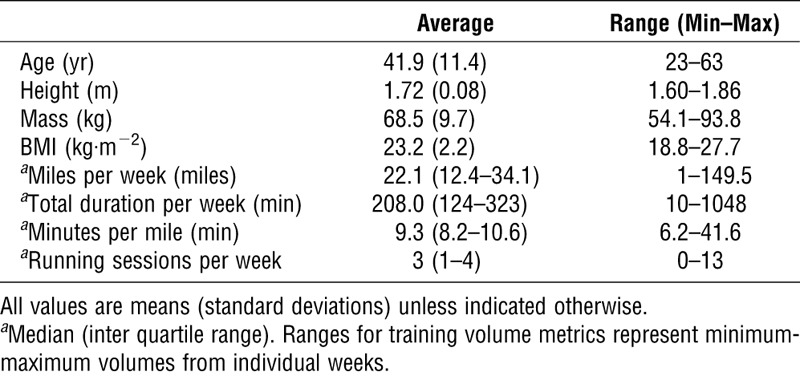
Summary characteristics for runners including self-reported weekly training volume metrics.

### Self-monitoring of Training Load

For the duration of the monitoring period, after each activity, runners were required to record the following data as soon as possible in a training log: date of session; start/end times (session duration calculated); activity/training type (e.g., road/off-road/track/treadmill run or other (e.g., gym, swimming, cycling, circuits or yoga)); running miles covered; an overall session RPE after consulting a visual scale, regardless of session type. Training logs were returned every 2 wk (mail) for manual input into a database. A composite measure of running training load (session RPE × session duration) in arbitrary units was subsequently calculated (7,24). Using activity/training type data, each day was classified as either a “running” (all surface types) or “nonrunning” day, with the latter further classified as either “other training” (e.g. gym, swimming, cycling, circuits or yoga) or a “rest” day. Where running training occurred twice on 1 d, running miles and duration were summed, a mean running RPE was calculated and training load was recalculated. If different types of training including running occurred on the same day, that day was labeled as a “running” day. Average self-reported weekly training load characteristics for the sample of runners are presented in Table 2.

### Accelerometer Monitoring of Training Load

Runners were issued with a GENEActiv accelerometer (100 Hz, triaxial, ±8*g*; Activinsights Ltd, Kimbolton UK) every other week to wear on their nondominant wrist to collect 7 d of data. Monitoring on alternate weeks allowed monitors to be refreshed and reduced participant burden to help maximize wear compliance during test weeks. Participants were requested to wear the monitor 24 h·d^-1^. As minimal differences exist in accelerometer output between monitors worn on dominant and nondominant wrists during higher intensity activity (28), runners were permitted to swap the wear location of the GENEActiv to their dominant wrist for the duration of a run if the wear location clashed with the preferred placement of another personal wearable device. Raw acceleration files were extracted and processed through an open-source package (GGIR Version 1.2–8, (26)) in R (http://cran.r-project.org) for autocalibration and calculation of the dynamic acceleration in milligravitational units (m*g*) averaged over 5-s epochs (the resultant vector magnitude corrected for gravity, ENMO, as described previously (27)). A total of 1532 d were obtained from which 1494 (97.5%) accelerometer days with at least 10 h of wear per waking day (29) were analyzed. Time accumulated in bins spanning 50-m*g* intervals between 50 and 4000 m*g* (50–99.99 m*g*; 100–149.99 m*g*; 150–199.99 m*g* etc) were obtained with activity <50 m*g* considered non–meaningful (21,30), and the incidence of time accumulated >4000 m*g* extremely brief and rare.

### Accelerometer Metrics and Statistical Analysis

#### Discriminating between “running” and “nonrunning” days (aim 1)

Accelerometer data were time-matched with training log data for each calendar day in STATA (version 15). Average Acceleration, Most Active 30-mins, and Mins≥400 m*g*, which are typical metrics used to describe characteristics of habitual PA, were considered candidates for discriminating between “running,” “other training” and “rest” days (Table 1). Receiver operating characteristic (ROC) analyses were carried out for these metrics to derive the optimum thresholds for discrimination between running and nonrunning training days. Performances were summarized by calculating the area under the ROC curves (AUROC). Similar to the methods by Evenson et al (31), thresholds were selected that optimized the balance between sensitivity (running classified as “running”) and specificity (nonrunning classified as “nonrunning”). Optimal thresholds were applied to the data and the percentage of days correctly classified as “running” and “nonrunning” calculated. The percentage of days correctly classified as “nonrunning” was further broken down according to whether the day was an “other training” day or a “rest” day. The percentage of misclassification for each type of “other training” misclassified as “running” was also identified. To detect a medium effect size with power of 80% and alpha of 0.05 (AUROC of 0.6 as significantly different from an AUROC of 0.5, no association), a total sample of at least 258 days (sample ratio of 1:1 with 129 positive days and 129 negative days) was required. The generalizability and performance of the ROC models on unseen data was assessed using leave-one-out-cross-validations (LOOCV) (32).

#### Estimation of external training load on accelerometer-classified “running” days (aim 2)

From training log data, miles, duration, and training load, which are frequently monitored to understand the influence of training load on performance, injury, and illness (1,3,6,7,24,33), were used to represent external and composite criterion measures of training load (criterion measures). On running training days that were classified using cut points from accelerometer metrics which demonstrated the highest levels of accuracy for correctly classifying running days (see aim 1), accelerometer-derived metrics of training load (Mins≥400 m*g* and WL400–4000 m*g*; Table 1) were examined to see how closely they corresponded to criterion measures. On each set of classified days, variances explained in training log criterion measures (miles, duration, and training load) by Mins≥400 m*g* and WL400–4000 m*g* were examined using linear regression analysis. The generalizability and performance of the model on unseen data was assessed using LOOCV. Statistical analyses were carried out in STATA (version 15) with an alpha level set at 0.05.

## RESULTS

### 

#### Discriminating between running and nonrunning days (aim 1)

From 35 participants, a total of 1494 d with >10-h wear were analyzed, of which 694 were “running” days, 641 were “rest” days, and 159 were “other training” days. Each participant contributed 18 to 56 d (mean [SD] = 42.7 [8.8]). Of these, 2 to 42 (19.8 [10]) were “running” days, 0 to 37 (18.3 [9.0]) were “rest” days, and 0 to 23 (4.5 [6.0]) were “other training” days.

Cutpoints for identifying running days from habitual PA using respective accelerometer metrics with area under curve (AUC) significant at *P* < 0.05 are presented in Table 3. Discrimination between “running” and “rest” days was excellent (88%–94% agreement; Table 3). Both the Most Active-30mins and Mins≥400 m*g* had >94% accuracy for classifying running as “running” and nonrunning as “nonrunning” and were subsequently used to separately classify running days for aim 2. “Average Acceleration” performed similarly for correctly classifying “nonrunning” days (93%), but was weaker at correctly classifying “running” days (88%). Irrespective of the metric, the greatest inaccuracy was from misclassifying “other training” days as “running” days, ranging from 14% misclassification for Most Active-30mins to 33% misclassification for Average Acceleration. The LOOCV procedure indicated robustness and stability as the high performance was maintained (AUC ≥0.93).

**TABLE 3:**
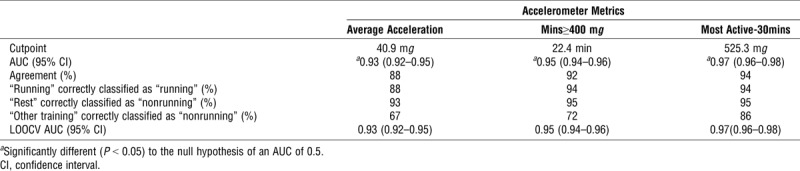
Optimum accelerometer cutpoints for differentiation between running and nonrunning days (includes rest days [no training] and other-training days [days with a different type of training]).

The rate of misclassification of other training activities as running is shown in Table 4. The most frequent other training activities undertaken were cycling (47 occurrences) and gym/exercise classes (45 occurrences). The most likely activities to be misclassified by the Average Acceleration metric were field/racket sport (95%), circuit training (57%), and then cycling (53%). A similar pattern was found when using Mins≥400 m*g*, except circuit training was not misclassified. The Most Active-30mins metric performed better for field or racket sports (25% misclassification) and generally across the board, but still misclassified nearly a third of cycling occurrences.

**TABLE 4:**
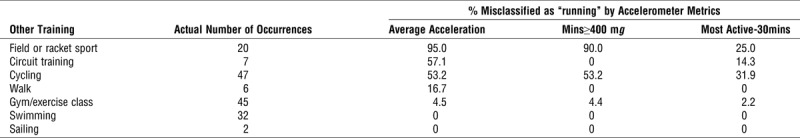
Percentage of “other training” activities misclassified as “running” when using “Average Acceleration,” “Mins≥400 m*g*”, and “Most Active-30mins” to discriminate between “running” and “nonrunning” days.

#### Estimation of external training load on accelerometer-classified running days (aim 2)

On running days classified using Mins≥400 m*g* or using Most Active-30mins (accelerometer metrics most successful at classifying running days from aim 1), the accelerometer-derived training load metric Mins≥400 m*g* explained approximately 75% to 76% and 74% of the variance in miles and duration, respectively, and 67% and 71% of the variance in training load (Table 5). The variance explained by WL400–4000 m*g* in miles, duration, and training load was slightly lower at 63% to 69% on running days classified using either Mins≥400 m*g* or Most Active-30mins, except for training load when running days were classified using Mins≥400 m*g*, which was much lower (55%). The LOOCV procedure indicated robustness and stability as the high performance was maintained in all cases.

**TABLE 5:**
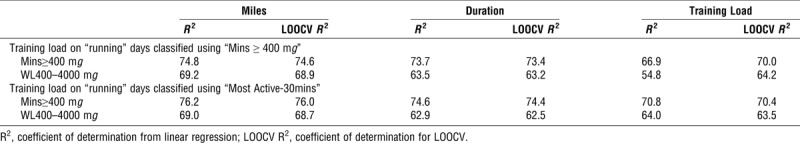
Percentage of the variance explained in miles, duration and training load when using “Mins≥400 m*g*” and “WL400–4000 m*g*” to quantify external training load on “running” days classified using Mins≥400 m*g* and Most Active-30mins.

## DISCUSSION

Raw acceleration data from wrist-worn accelerometers widely used in research can be used to objectively, unobtrusively, and accurately identify running training days and quantify external training load in runners. Importantly, the accelerometer metrics used are embedded within existing, validated open-source software for processing and analyzing accelerometer data for accurate quantification of habitual PA (26,27). As a field-based proxy measure of external mechanical load (19,20), use of these accelerometer-derived metrics will enhance future research that seeks to further understand the influence of objectively measured modifiable patterns of external training load relative to rest and sleep on RRI and performance outcomes (1–8).

### 

#### Discriminating between running and nonrunning days

The high degree of accuracy for correctly classifying running days and days with no training indicates that wrist-worn accelerometer metrics can be used to objectively and unobtrusively discriminate between running and nonrunning days. While each accelerometer metric was able to discriminate between these days, the mean acceleration recorded during “Most Active-30mins” was the best discriminator. As running is characterized by high accelerations (23), which incorporate an impact peak (19,20), high accelerations for the most active continuous 30 min of the day likely reflect the deliberate inclusion of a running session. The length of the training session may not match 30 min, but the elevation of the acceleration alone is sufficient to simply differentiate between running and nonrunning days. In contrast, metrics that sum time spent at high accelerations across the day (Mins≥400 m*g*), or the average accelerations across the day, can be elevated due to short activity bursts spread across the day which may or may not be part of a training session.

Even for the Most Active-30mins, a degree of misclassification in “field or racket sports” and “circuits” is likely due to these activities, including aspects of running or lunging and jumping, which could elevate average acceleration to exceed magnitudes typically found during running (19,20). For cycling, road or track vibration also has the potential to elevate this average acceleration to similar levels found when running. *Post hoc* analysis of demographic and training data indicated that very short runs may be a potential source of misclassification. However, we are also cognizant that validation of accelerometer data in the field is complicated by the use of potentially inaccurate self-reported training log or training program information (underreported and overreported training activity) (9,10). In using this simple metric to identify running training days in future studies, any accepted level of misclassification will depend on the nature of the activity misclassified relative to the research question.

#### Estimation of external training load on running days

When running days were classified using Most Active-30mins, approximately 71% to 76% of the variance in Miles, Duration or Training Load was explained by the accelerometer-derived training load metric Mins≥400 m*g*, which was approximately 7%–13% more than the variance explained by the composite workload metric WL400–4000 m*g* on these days. When running days were classified using Mins≥400 m*g*, similarly high levels of variance in miles and duration were explained by the accelerometer-derived training load metrics Mins≥400 m*g* and WL400–4000 m*g* compared with when days were classified using Most Active-30mins but 4% and 9% less variance was explained in training load by respective metrics on these days. Despite differences, these accelerometer-derived metrics correspond highly with criterion measures, especially miles and duration, which suggests a high degree of convergent validity with existing training log methods for quantifying external training load. Mins≥400 m*g* in particular appears to be a good measure of external training load and can be easily obtained from longitudinal monitoring of habitual PA. For comparison, fast walking at 5 km·h^−1^ in adults yields approximately 170 ± 56 m*g* from a wrist-worn accelerometer, whereas running at 8 km·h^−1^ yields approximately 760 ± 200 m*g* (23). A threshold of 400 m*g*, which is also validated to quantify vigorous activity equivalent to 6 METs (23) (Table 1), therefore, provides sufficient margins to avoid capturing lower-intensity walking-type activity while capturing lower accelerations introduced by large variability when running at 8 km·h^−1^ and lower accelerations from slower speed running. For comparison, additional analysis of accelerometer-derived metrics of external training load on days classified using training log information, indicated that Mins≥400 m*g* (75%, 75%, and 72%, respectively) and WL400–4000 m*g* (65%, 60%, and 62%, respectively) explained similar variation in miles, duration and training load when days were classified using either Mins≥400 m*g* or Most Active-30mins. An ability to use a single simple accelerometer-derived metric (e.g., Mins≥400 m*g*) to accurately classify running days and provide a valid measure of external training load, lays the foundation for overcoming challenges, such as ease of use and data interpretation described by Bourdon and colleagues (5) for accelerometry to be used in training program prescription. Further, it would be possible to use the regression analyses to predict outcomes familiar to runners (e.g., miles), but for analytical purposes, we believe it preferable to use the directly measured metrics.

Accelerometer metrics are also highly correlated with laboratory-based measures of ground reaction force (19,20), which suggests that accelerometer-derived metrics of external training load may add more value to models of RRI and performance than existing training log-based measures. Further research to determine whether Mins≥400 m*g* and/or WL400–4000 m*g* translate into meaningful measures of external training load in relation to injury and performance would be beneficial.

#### Implications of this study

The ability to obtain accurate, objective training records without the need for user input removes the reliance on the creation of a subjective training log, reduces participant burden, avoids bias, and other reporting inaccuracies associated with logging or marking data on paper or a device, (9,10) and facilitates the accurate monitoring of runners’ training behavior in future prospective studies. It also removes the need to match training log data, sometimes with multiple entries, with accelerometer data across days. A high monitor wear compliance (90% of days >16 h; 76% of days >22 h) in this population also supports its inclusion in the future analysis of patterns of training relative to rest and sleep. In contrast to GNSS devices, which are reliant on tracking a physical change in position in an outdoor environment (5), accelerometers also have the advantage of being able to be used anywhere, even to monitor external load when running on the spot. Developing accelerometer-derived measures of external training load provides a natural extension of an accelerometer’s existing ability to accurately measure all aspects of habitual PA including rest and sleep longitudinally.

#### Further developments

Streamlining methods for collecting, generating, and visualizing simple accelerometer-derived training load metrics (5) could facilitate their inclusion in commercial activity trackers and healthcare monitors for training program monitoring, prescription, and injury prevention purposes. It would also be beneficial to examine time spent at higher intensities of acceleration (e.g. >1000 m*g* approximating 10 km·h^−1^; 28) to separately analyze higher and lower-intensity running. In an effort to avoid bias from self-reported measures of miles and duration (10), further comparison of accelerometer-derived metrics with objectively measured criterion measures (e.g., GPS) might be beneficial, however it would be important to avoid over-burdening the runner with a requirement to wear multiple monitoring devices. To improve the classification of running from other training activities, alternative analysis methods such as those used in the sedentary sphere (34,35), which consider the orientation of the monitor due to wrist position to estimate upright, sitting, and lying down postures, could also be explored to distinguish running from other activities, such as cycling. Analysis of the frequency compositions of the raw acceleration signal from different activities may also allow the selection of suitable filters to improve classification performance or enable metrics to be developed that allow cycling and racket sports to be identified explicitly. Although the high performance from the LOOCV carried out in this study demonstrate the robustness of using accelerometer metrics to classify running days, further validation of the method in an independent sample would also be beneficial.

## STRENGTHS AND LIMITATIONS

We used longitudinal methods for objectively monitoring habitual PA in runners every other week and obtained written self-reported training logs every week over at least a 9-wk period. The incorporation of runners leading up to the events of differing lengths ensured a wide range of training patterns for testing. The results were robust across this range. During this period, runners remained motivated to complete training logs and were familiar with wearable devices yielding a large number of matched training log and accelerometer days with the added benefit of high accelerometer wear compliance. A rich bank of data was therefore obtained, allowing robust statistical methods to be used with cross-validations to address each research question. However, the nature of the sample does limit the generalizability of the results. All participants were self-identified runners who were training for an event. Most did undertake some form of cross-training, but the degree of engagement in other activities may be greater in people who do not identify as runners, or runners when they are not leading up to an event. Further research should investigate the degree of misclassification of “other training” as “running” in other populations.

## CONCLUSIONS

Wrist-worn accelerometer metrics can be used to objectively, unobtrusively, and accurately identify running training days in runners, reducing the need for training logs or user input in future prospective research or commercial activity tracking. A high percentage of the variance explained in existing metrics by new, simple, accelerometer-derived metrics of external training load supports the development and future use of accelerometry for prospective, preventative, and prescriptive monitoring purposes in runners.

## Supplementary Material

SUPPLEMENTARY MATERIAL
