# Supplementary material for: Wrist-worn Accelerometry for Runners: Objective Quantification of Training Load
Source: Med Sci Sports Exerc. 2018 Jul 30;50(11):2277–84. doi: 10.1249/MSS.0000000000001704 (PMC6195805; doi:10.1249/MSS.0000000000001704)
Supplement: SUPPLEMENTARY MATERIAL [file mss-50-2277-s001.docx]

**Equivalent Hypertrophy and Strength Gains in HMB- or Leucine-supplemented Men**

Josephine S. Jakubowski^1^, Edwin P.T. Wong^1^, Everson A. Nunes^2^,

Kenneth S. Noguchi^1^, Joshua K. Vandeweerd^1^, Kevin T. Murphy^1^,

Robert W. Morton^1^, Chris McGlory^1^, and Stuart M. Phillips^1^

^1^Department of Kinesiology, McMaster University, Ontario, Canada; ^2^Department of Physiological Sciences, Federal University of Santa Catarina, Florianopólis, Brazil

**Running title:** Equivalent gains with HMB or leucine

**Author for correspondence**

Prof. Stuart Phillips, Ph.D.

Department of Kinesiology, McMaster University,

1280 Main Street West, Hamilton, Ontario, Canada

T: +1-905-525-9140 x24465

E: phillis@mcmaster.ca

The authors declare that the results of the study are presented clearly, honestly and without fabrication, falsification or inappropriate data manipulation. The results of the present investigation do not constitute endorsement by ACSM. SMP declares that he has received competitive grant funding, honoraria, and travel expenses from the US National Dairy Council. No other authors declare any conflict of interest. This work was supported by internal McMaster University grant funds. EAN has received a grant from the Brazilian National Council for Scientific and Technological Development (CNPq) (Science without borders program) and is a CNPq Research Productivity Fellow. SMP receives grant funding from the National Science and Engineering Research Council of Canada and the Canadian Institutes for Health Research as well and the Canada Research Chairs program and acknowledges those sources of support during this investigation.

**ABSTRACT**

Ingestion of proteins with high leucine content during resistance training (RT) can augment hypertrophy. Some data suggest that a leucine metabolite, β-hydroxy, β-methylbutyrate (HMB), is substantially more anabolically efficacious than leucine. **Purpose:** We aimed to test whether supplementation with HMB versus leucine, added to whey protein, would result in differential muscle hypertrophy and strength gains in young men performing resistance training. **Methods:** Twenty-six resistance-trained men (23 ± 2 y) performed 12 wk of RT with 3 phases. Phase 1: 8 wk of periodized RT (3 training sessions/wk). Phase 2: 2 wk overreaching period (5 sessions/wk). Phase 3: 2 wk taper (3 sessions/wk). Participants were randomly assigned to twice daily ingestion of: whey protein (25 g) plus HMB (1.5 g) (Whey+HMB; n=13) or whey protein (25 g) plus leucine (1.5 g) (Whey+Leu; n=13). Skeletal muscle biopsies were performed before and after RT. Measures of fat and bone-free mass (FBFM), *vastus lateralis* (VL) muscle thickness and muscle cross-sectional area (CSA – both by ultrasound), muscle fiber CSA, and 1-repetition maximum (1-RM) strength tests were determined. **Results:** We observed increases in FBFM, VL muscle thickness, muscle CSA and fiber type CSA and 1-RM strength with no differences between groups at any phase. We observed no differences between groups or time-by-group interactions in hormone concentrations at any phase of the RT program. **Conclusion:** HMB added to whey did not result in greater increases in any measure of muscle mass, strength, or hormonal concentration compared to leucine added to whey. Our results show that HMB is no more effective in stimulating RT-induced hypertrophy and strength gains than leucine.

Key words: periodized resistance training, resistance training, β-hydroxy-β-methylbutyrate

**INTRODUCTION**

Resistance training (RT) is an effective strategy for athletic and clinical populations to enhance muscle size and strength (1). To maintain muscular fitness, the American College of Sports Medicine (ACSM) recommends that healthy adults should engage in RT a minimum of two days per week (2). In addition to positive effects of RT on muscle mass accretion, the ingestion of high-quality protein has an additive effect on gains in mass and strength (3). A recent meta-analysis from our group showed that protein supplementation augmented gains in muscle mass and strength with daily protein intakes of 1.6 g/kg body mass/day being a plausible upper limit for augmentation of lean mass accretion and strength gains (3). The anabolic influence of protein supplementation on skeletal muscle mass is likely related to leucine content (4). Whey protein is acid soluble, rapidly digested, and contains a high proportion of leucine (4). Ingestion of leucine alone can independently stimulate the mechanistic target of rapamycin complex-1, which is a key signaling protein important in activating muscle protein synthesis (MPS) (5, 6).

A leucine metabolite β-hydroxy-β-methylbutyrate (HMB) exerts similar effects on skeletal muscle in terms of stimulation of MPS (5) and can augment increments in RT-induced muscle mass and strength (5, 7-11). Some reports have demonstrated considerable [well above normal (3)] RT-induced increases in lean body mass and strength with supplementation of HMB as a free-acid (HMB-FA) (10, 11) in trained young men, as well as with supplementation with the calcium-salt of HMB (HMB-Ca) (7). These data (7, 10, 11), suggest an unprecedented potency to HMB to augment RT-induced gains in strength and muscle mass well beyond protein and/or leucine. Interestingly, the addition of 400 mg of ATP to HMB-FA (11) appeared to increase gains in muscle mass even further than HMB-FA alone; however, this study has been challenged on methodological grounds (12).

Although data exist supporting substantial anabolic properties of HMB-FA (10, 11) and HMB-Ca (7), the increases in lean body mass and strength observed in these reports are difficult to reconcile with extant knowledge of what is typical of RT-induced hypertrophy. For example, including only young participants (<35y; n=624) from our recent meta-analysis with a mean study duration of ~12±6 wk, the gains in lean body mass from RT averaged 1.2 ± 1.1 kg (3) and protein supplementation resulted in only an additional 0.4 kg increase. The gains in fat- and bone-free mass (FBFM; i.e., lean body mass) reported by Kraemer et al (7), Wilson et al (10) and Lowery et al (11) were ~9 kg, 7.4 kg and 8.5 kg FBFM, which are, respectively, 5.6, 4.6, 5.3 times greater than the mean ~1.6kg gain (see above) we reported in protein-supplemented young men (3).

In previous investigations, the placebo comparator to HMB-FA was simply carbohydrate (10, 11). In another investigation (7), the HMB supplement also contained other ingredients while the placebo was simply maltodextrin (7). Here, the aim was to conduct a randomized double-blind pragmatic trial of supplementation with whey protein with HMB-Ca, as this supplement was previously reported to result in the greatest gains in FBFM (7), versus whey protein plus the parent compound of HMB, leucine. We assessed muscle hypertrophy using multiple indices and strength while employing a highly effective program of undulating periodized RT (7, 10, 11) in young relatively trained men. In line with previous investigations (10, 11), we hypothesized that whey protein enriched with HMB would elicit substantially superior gains in lean mass and strength compared to whey protein enriched with leucine.

**METHODS**

**Participants.** Participants were 26 recreationally trained (twice/week RT) men participated in the study. Participants were: non-smokers, free of prescription/non-prescription medication, were active RT men and healthy according to the Physical Activity Readiness Questionnaire (PAR-Q). Baseline characteristics of the participants can be found in Table 1. Of the 30 participants recruited, one dropped out as a result of an injury sustained during the RT program, one due to an injury sustained in a non-intervention related event, one relocated, and one did not adhere to the training or supplement program leaving us with n=13 per group (see Figure, Supplemental Digital Content 1, Flow chart of subject recruitment and group allocation, http://links.lww.com/MSS/B378). The final sample size was, based on previous data (7, 10, 11), more than adequate to detect an impact of HMB, if one existed, based on effect sizes seen in these studies.

Participants were informed of the purpose, experimental procedures and risks before providing informed written and verbal consent. The study was approved by the Hamilton Integrated Research Ethics Board and was conducted in accordance with the Canadian Tri-Council Policy on the use of human participants in research. The trial was registered at <https://www.australianclinicaltrials.gov.au/> as 12616000197437.

**Study Design.** A schematic overview of the study design is shown in (see Figure, Supplemental Digital Content 2, Schematic of study design, http://links.lww.com/MSS/B379). We employed a randomized, double-blind repeated measures design. A third independent party performed the randomization and assignment codes were not revealed to study personnel or participants until all data had been analyzed. Participants were randomized, according to a list generated at randomize.com, with block sizes varying from 2-6 matched for baseline FBFM, to ingest: whey protein (25 g) plus HMB as its calcium salt (1.5 g: Whey+HMB, n=13) or whey protein (25 g) enriched with leucine (1.5 g: Whey+Leu; n=13), twice daily during a 12-week, 3-phase RT program as described extensively previously (7, 10, 11). The RT program was selected as it has been previously demonstrated to result in substantial gains in muscle mass and strength (7, 10, 11) and to elicit muscle damage in trained participants when HMB is hypothesized to be maximally effective (7, 10, 11, 13). Briefly, phase 1 was 8 weeks of undulating periodized RT thrice weekly; phase 2 was an overreaching phase (5 weekly training sessions); followed by phase 3, which was a two-week taper (3 sessions/week). Body composition and 1-RM strength tests were performed at baseline, weeks 4, 8, 9, 10, and upon completion of the RT program (week 12) as illustrated in Supplemental Digital Content 2 (see Figure, Supplemental Digital Content 2, Schematic of study design, http://links.lww.com/MSS/B379). Supplements were prepared by Infinit Nutrition (Windsor, ON, Canada) matched for flavor (citrus) and consistency and were in powder form, which dissolved freely into 250 mL of water. A sample of the supplement was analyzed by a third party LGC (ISO17025 accredited; Teddington, UK) for compounds specified within the Service Level Agreement for Nutritional Supplements V2.0 with GCMS and LCMS with no results found (LGC Reference: 204545). Third-party testing (Labs-Mart Inc. Edmonton, AB) of the same supplements (reports 70424-2 and 70424-1) for protein, leucine and HMB indicated the supplements contained 98±1% of the protein, 98±1% of the free leucine, and 97±1% of the HMB as prepared. On training days, supplements were consumed following training and prior to sleep. On non-training days, supplements were consumed in the morning and prior to sleep.

**Familiarization and 1-RM strength testing.** One week prior to the start of the training program participants attended a familiarization session at a research dedicated training facility located within McMaster University, and ≥ 72 hours later (14), a 1-RM strength test for squat, bench press and deadlift. The 1-RM tests were performed in the same order: squat, bench press and deadlift and followed strict guidelines as established by the National Strength and Conditioning Association (NSCA). One investigator conducted all strength tests. Strength tests began with a 5-min warm-up on a cycle ergometer, that was followed by 5-8 repetitions at 50 %, followed by 3-5 repetitions at 75 % of the predicted 1-RM load. Following 5 mins of seated rest the 1-RM load was increased by 10-20 % until 1-RM was achieved. Participants had their form critiqued and adjusted if necessary by qualified personal trainers. Total 1-RM strength was calculated as the sum of mass lifted (kilograms, kg) for 1-RM of squat, bench press and deadlift.

**Dietary Records.** Protein intake was assessed at weeks 0, 8, and 12, using a 3-day food diary (2 weekdays, 1 weekend day), and was analyzed using the NutriBase dietary analysis software (Nutribase11 Professional Edition, version 11.5, Cybersoft Inc., Phoenix, AZ, USA). Food diaries were completed on both training and non-training days.

**Resistance Training Intervention**. Replicating a program described extensively elsewhere, (7, 10, 11), participants engaged in a supervised 3-Phase RT program. Our program followed this program exactly with participants performing: Squat, bench press, deadlifts, dumbbell shoulder press, pull-ups/dips, bent over row, biceps curls/lying triceps extensions, with leg press and close-grip bench press performed in weeks 9 and 10. Loads were decreased (5-10%) between sets to ensure participants achieved the prescribed repetition ranges.

**Body composition.** Body composition was assessed using dual- energy x-ray absorptiometry (DXA; GE Lunar iDXA total body scanner, GE Medical Systems, Madison, WI, USA) between 06:00-09:00 following a ≥10 hour fast and after participants had voided their bladders. Participants refrained from physical activity for ≥24 hours except during Phase 2 (training 5 x/week). Scans were performed and analyzed with software in the medium scan mode. Total body water was assessed using a Bioelectrical impedance analysis (BIA, BIA-101A; RJL Systems, Mt. Ckemens, MI). All body composition assessments were performed by the same technician to minimize variability. Based on scans of a whole-body phantom (Oscar Jr. Orthometrix, Naples, FL), intra-assay coefficient of variation (CV) of this scanner for FBFM (i.e., the body compartment of interest) is 1.2% and less than 1.7% on inter-assay 12 wks apart.

**Ultrasound muscle thickness and cross-sectional area.** Muscle thickness (MT) of the *vastus lateralis* was assessed by the same investigator using a B-mode ultrasound (Vivid q; GE Medical Systems, Horten, Norway) and a 50 mm, 12.5 linear-array probe. Ultrasound assessments were performed fasted and at the same time as the body composition testing. Participants laid supine for 10 mins with their right leg in full extension in a custom mount. Thickness was assessed at fifty percent of the distance between the greater trochanter and the lateral epicondyle of the knee (15). Tracing paper was used to record the reference point and the probe was placed transversally on the leg. An experienced investigator used water-based gel to ensure good acoustic contact and applied no pressure to the skin to rule out tissue compression as a potential confounding influence (16). A second investigator ensured the images were clear and possessed identifiable superficial/deep aponeurosis and the MT image was stored. To assess muscle cross-sectional area (CSA) sequential images starting at the border of the rectus femoris to the border of the biceps femoris in the frontal plane were captured resulting in a total of ~8-10 images of the *vatus lateralis* (17). Images were stored using Echo-PAC, PC Version 110.0.2 (GE Medical Systems, Horten, Norway) and converted from DICOM to JPEG using Sante DICOM Editor (Version 3.1.20; Santesoft Athens, Greece). The MT images were analyzed using AMS II (Version 1.141; Gothenburg, Sweden) at the widest distance between the narrow and deep aponeurosis. Each image was reviewed, and manual corrections were made, if necessary, the algorithm redirected the borders to asssses the thickness accurately (18). The same investigator performed the MT analysis on AMS II software on two separate occasions (intra-class correlation coefficient = 0.96). The muscle CSA images were stitched together using GIMP (GNU Image manipulation program 2.8.22, Creative Common, Mountain View, CA, USA) by aligning the superficial and deep aponeuroses. The muscle CSA was measured using computerized planimetry (i.e., VL muscle CSA was manually contoured with an 800-dpi mouse) (Madena 3.2.5, EyePhysics, Los Paladinos, USA). The planimetry software was calibrated with fixed distance scales displayed in the ultrasound images. The ultrasound-based technique for determination of muscle CSA has recently been validated against magnetic resonance imaging (17). The stitched images were downloaded to ImageJ and manually traced to encompass the entire *vastus lateralis.* Conversion factor of 69 pixels per cm was used to calculate CSA in cm^2^. Two experienced investigators performed the analysis with an interclass correlation of 0.97.

**Power testing**. *Wingate.* Participants performed a 2-minute warm-up (50 watts, W) on an electronically braked cycle ergometer (Veletron, RacerMate, Seattle, WA, USA). Participants were instructed to pedal as fast as possible against the ergometers initial resistance for approximately 2 seconds before the appropriate load was applied by a computer interfaced with the ergometer (Wingate software version 1.11, Lode). Similar to previous investigations (19), a 30-second “all out” effort against a resistance equivalent to 0.075 kg/kg body mass was completed. The total body mass used for all power assessments was derived from the DXA. Participants were verbally encouraged throughout the test. Research assistants were trained at the same time and provided with a standardized set of verbal cues to encourage participants. Peak power, mean power and fatigue index were recorded. Participants then engaged in a 5-minute dynamic cool down.

*OptoJump*. Measurements of specific power (W/kg) were made using OptoJump (Microgate, Via Antonio Stradivari, Italy) and analyzed using OptoJump Next systems software (Microgate, Via Antonio Stradivari, Italy). Participants performed one practice countermovement jump to ensure correct form and completed three countermovement jumps. Data were averaged as specific power (W/kg).

**Muscle Fiber and Cross-sectional area.** Participants arrived overnight fasted and 72h after their last training session for a muscle biopsy. Muscle biopsies were obtained at baseline and post-training from the *vastus lateralis.* Muscle biopsies were performed under local anesthesia (2% xylocaine) using a 5-mm Bergstom needle that was modified for manual section. Upon excision, the sample was cleared of connective tissue and fat and was oriented longitudinally before being embedded in an optimal cutting temperature medium. The mounted sample was frozen in liquid isopentane, cooled by liquid nitrogen and stored at -80°C for analysis. Cross sections (7 μm thick) were cut on a Microm HM550 Cryostat (Thermo Fisher scientific, Waltham, MA, USA), mounted on glass slides and stained. Fiber type and CSA were assessed via immunofluorescent staining of myosin heavy chain (MHC) isoforms and dystrophin as previously described (20). Primary antibodies against MHC1 (BA-F8), MHC11A (SC-71), MHC11X (6H1) and dystrophin (MANDYs; Developmental Studies Hybridoma Bank, Iowa City, IA, USA) followed by isotope-specific fluorescent secondary antibodies for the identification of Type I, Type I /Type IIA, Type IIA, Type IIA/X and Type IIX fibers. Slides were mounted with Prolong Diamond Antifade Reagent (Life Technologies, Burlington, ON, CAN) and imaged the next day. Images were obtained with a Nikon Eclipse 90i microscope at a magnification of 20X and captured with a Photometric Cool SNAP HQ2 florescent camera (Nikon Instrument, Melville, NY). Analysis was performed using the Nikon NIS element AR software (Nikon Instruments) on a large-scale image. The investigator was blinded to the group and time condition of each participant during all analyses. The CSA data pools Type IIA and Type IIX fibers as Type II, due to the number necessary to analyze the CSA for each fiber type (~50-60) per sample (21). Fiber type was assessed by counting all suitable fibers (mean fibres counted: 239 ± 71) whereas fibers along the periphery and those that were obliquely or longitudinally-oriented were not included.

**Blood Analysis.** Blood samples were obtained to measure creatine kinase and systemic hormones concentrations. A 22-gauge needle was inserted into an antecubital vein and blood was collected and set aside to be allowed to clot and serum was collected after a 10-min spin at 500 *g,* Heparinized-tubes were used to isolate plasma. Whole blood samples were immediately analyzed for creatine kinase (ARCHITECT System, Abbott Laboratories, Abbott Park, IL 60064, USA) while the remaining samples were centrifuged at 4000 g for 10 min at 4° C, aliquoted and frozen at -80℃. Blood samples were analyzed for serum total testosterone (T; ng/dL), free testosterone (fT; ng/dL), cortisol (nM), growth hormone (GH; ng/mL) and insulin-like growth factor 1 (IGF-1; ug/dL) using a two-site chemiluminescence immunometric assays (Immulite; Intermedico, Holliston, MA) or radio-immunoassay (Diagnostics Products Corporation, Los Angeles, CA). The intra- and inter-assay CV for these hormones were all below 5%.

**Statistical analyses.** All variables were assessed for normality using the Kolmogorov-Smirnov test (p>0.05). Baseline characteristics were assessed using independent-t tests. A two-way (group by time) mixed-model ANOVA was assessed for muscle thickness, fibre CSA, fibre distribution and total 1-RM strength and hormone concentrations. Tukey’s HSD was performed in excel when the ANOVA was significant. Significance was set at p<0.05. To compare the changes in body composition (FBFM, MT, CSA) and strength tests (1-RM, Wingate peak power and Optojump) between groups independent t-tests were performed. Intra-class correlation estimates for muscle thickness were calculated based on a single rater, absolute-agreement, 2-way mixed-effects model. The interclass correlation for muscle cross-sectional area was assessed using a mean-raters (k=2), consistency-agreement, 2-way mixed-effects model. All analysis was performed using SPSS statistical package version 23 (IBM SPSS Statistics for Windows, Version 23.0. Armonk, NY). Values are expressed as means ± standard deviation (SD) or using box and whisker plots to illustrate the full variance of the data.

**RESULTS**

**Participant Characteristics.** Baseline anthropometric characteristics are provided in Table 1. There were no significant differences between groups for any study variables (p>0.05). There was no significant difference in dietary protein intake (g/kg/day) between groups at baseline or week 12 (p>0.05, Table 1).

**Body composition.** FBFM changed in the Whey+HMB from 63.1 ± 5.7 kg to 65.4 ± 5.9 kg. For Whey+Leu, FBFM changed from 62.9 ± 8.7 kg to 65.6 ± 8.6 kg, pre to post-invention, respectively (Figure 1A). The change in FBFM for Whey+HMB (2.3 ± 1.2 kg) and Whey+Leu (2.6 ± 1.9 kg) were not significantly different (p=0.59; Figure 1B). Fat mass remained unchanged (p=0.19) in both groups (Whey+HMB: -0.1 ± 0.9 kg and Whey+Leu: -0.5 ± 1.3 kg; p=0.41). For both groups, total body water remained unchanged throughout the intervention (p=0.62, data not shown).

**Ultrasound.** Whey+HMB and Whey+Leu exhibited comparable changes in MT. Whey+HMB increased from 31 ± 2 mm to 32 ± 2 mm and Whey+Leu increased from 30 ± 3 mm to 32 to 4 mm (5 ± 6 % and 5 ± 6 %, respectively; p=0.97, Figure 2A). The mid-thigh CSA increased from 34.1 ± 4.0 cm^2^ to 36.4 ± 4.8 cm^2^ in Whey+HMB and from 33.9 ± 5.8 cm^2^ to 36.1 ± 6.3 cm^2^ in Whey+Leu (Figure 2C). The change in muscle CSA for Whey+HMB (2.2 ± 1.4 cm^2^) and Whey+Leu (2.3 ± 1.4 cm^2^) was not different (p=0.96, Figure 2D). The percent for change in CSA for Whey+HMB (6 ± 4 %) and Whey+Leu (7 ± 4 %) in CSA was similar between groups (p=0.92).

**Fiber CSA.** Following the intervention, there was an increase in Type 2 CSA with no difference between groups or fibers (p>0.05, Table 2). There were no group, time or group-by-time interactions for Type 1 or Type 2 fiber-type distributions (p>0.05). A significant shift pre- to post-training in Type 2x from 11 ± 9 % to 1 ± 2 % in Whey+HMB and 6 ± 8 % to 4 ± 7 % in Whey+Leu; p=0.03, Table 2) was observed.

**Maximal Strength.** In response to 12 weeks of RT, 1-RM strength for squat, bench press and deadlift increased (p<0.01). There was an increase post-training in squat 1-RM for Whey+HMB (33 ± 10 kg) and Whey+Leu (35 ± 17 kg, Table 3). The increase in bench press 1-RM was similar for Whey+HMB (11 ± 5 kg) and Whey+Leu (11 ± 7 kg, Table 3). The increase in deadlift 1-RM for Whey+HMB (25 ± 12 kg) was similar to Whey+Leu (34 ± 22 kg, Table 3). There were no significant differences in any 1-RM strength test from baseline to week 12 between for Whey+HMB and Whey+Leu (p>0.05). Following 12 weeks of training, there was a significant increase in total strength for Whey+HMB (70 ± 21 kg) and Whey+Leu (80 ± 40 kg) with no significant difference between groups (p=0.41). Upon completion of week 1 to week 8 (Phase 1) both groups experienced similar changes in squat 1-RM (21± 11 kg and 27 ± 11 kg, p=0.24), bench press (10 ± 4 kg and 10 ± 5 kg, p=0.89) and deadlift (18 ± 7 kg and 25 ± 19 kg, p=0.22) for Whey+HMB and Whey+Leu respectively. Upon completion of Phase 2 (overreaching), both groups experienced similar changes in squat (3 ± 7 kg and -2 ± 9 kg, p=0.24), bench press (-2 ± 4 kg and -1 ± 3 kg, p= 0.43) and deadlift (-2 ± 8 kg and -6 ± 11 kg, p=0.15), Whey+HMB and Whey+Leu, respectively. ~~Following overreaching, the percent change in 1-RM strength for squat (2 ± 4 %, -1 ± 5 %) bench press (-1 ± 4 %, -1 ± 3 %) and deadlift (-2 ± 5 %, -3 ± 7 %) was similar for Whey+HMB and Whey+Leu, respectively~~. The change in total strength following week 8 to week 10 (overreaching) was 1 ± 12 kg for Whey+HMB and 8 ± 19 kg for Whey+Leu. (p=0.29). Following a 2-week taper (Phase 3), total 1-RM strength recovered similarly between groups (p=0.25) from overreaching: Whey+HMB (20 ± 9 kg) and Whey+Leu (28 ± 22 kg). There was a significant increase in total 1-RM strength from (Week 8 to Week 12) with no difference in Whey+HMB (21 ± 10 kg) and Whey+Leu (18 ± 18 kg, p=0.59).

**Wingate.** There was a significant increase in peak Wingate power (W) following training for Whey+HMB (981 ± 180 W to 1030 ± 180 W) and Whey+Leu (954 ± 90 W to 1043 ± 109 W, p=0.02) with no difference between groups (p=0.39, Table 3). The delta change in peak power was not different between groups (p=0.33), 49 ± 96 W and 88 ± 92 W for Whey+HMB and Whey+Leu, respectively. There was a significant group-by-time interaction in peak power following overreaching (Week 8 to Week 10) for Whey+HMB and Whey+Leu (p=0.03). The decrement in peak power for Whey+HMB was significantly greater (-39 ± 74 W) than Whey+Leu (18 ± 51 W) (p=0.03).

**Optojump.** Following training, there was a significant increase in Optojump performance (W/kg); Whey+HMB increased from 14.9 ± 1.3 W/kg to 16.1 ± 1.3 W/kg, and Whey+Leu increased from 14.4 ± 1.9 to 15.7 ± 1.9 W/kg (p<0.01), with no difference between groups (p=0.80). There was no change in average power (W/kg) during overreaching for Whey+HMB (15.2 ± 1.3 W/kg to 15.9 ± 1.5 W/kg) or Whey+Leu (16.0 ± 3.0 W/kg to 16.5 ± 1.9 W/kg, p=0.80).

**Blood Analysis.** There was a significant increase in blood creatine kinase activity from baseline and week 8 to week 9, and week 10 (p<0.05), with no significant differences between groups (Table 4). Following overreaching, CK increased 109 ± 115 % and 72 ± 41 % (p=0.29, Table 4) for Whey+HMB and Whey+Leu, respectively. Following a 2-week taper (week 10 to week 12) both groups experienced similar decrements in CK -26 ± 44 % and -29 ± 57 %, p=0.91 (Table 4, Whey+HMB and Whey+Leu, respectively). There was a significant increase in cortisol from baseline and week 8 to week 9, week 10 (overreaching) and week 12 (p<0.01), with no difference between groups. Both groups showed similar increases following overreaching (47 ± 49 % and 47 ± 43 %, p=0.99) and a two-week taper (-15 ± 34 % and -10 ± 48 %, p=0.74) for Whey+HMB and Whey+Leu, respectively (Table 4). There was no time or group by time effect for IGF-1 (p=0.14). There was no significant group by time effect for GH (p=0.42) but a significant effect of time (p=0.017). In the Whey+HMB and Whey+Leu groups, there was a significant decrease in GH from week 0 to week 10 and week 12 (p<0.05). There was a significant decrease (p≤0.05) in total testosterone from week 0 to week 10, and a significant increase from week 10 to week 12 (p<0.05) with no between-group differences at any phase. There was a significant increase in free testosterone from baseline to week 8 (p<0.05) and a significant decrease from week 8 to week 10 (p=0.05).

**DISCUSSION**

We discovered that in young men undertaking a periodized undulating RT program, that the ingestion of whey protein (50g) with HMB-Ca (3 g daily) versus whey protein with the same amount of leucine (3 g daily) resulted in no differences in training-induced gains in FBFM, muscle size, strength, or power. We did not detect differences in blood hormones and serum creatine kinase, an often-used proxy marker of muscle damage. Thus, contrary to our initial hypothesis, and in direct contradiction to several reports using either HMB-Ca (7) or HMB-FA (10, 11), we conclude that HMB ingestion does not lead to an enhancement of hypertrophy or strength. We observed similar increases in DXA-derived FBFM for Whey+HMB (2.3 ± 1.2 kg) and Whey+Leu (2.6 ± 1.9 kg). We also assessed total body water content at the time of the DXA scan and found no differences between each participant’s scans that would have accounted for changes in FBFM. The DXA-measured changes in FBFM we observed were ~3-3.7 times *lower* than the gains previously reported (7, 10, 11). We cannot explain why the gains in FBFM we report are so much lower than these previous studies (7, 10, 11), but suggest that numerous methodological issues may contribute to the discrepancies (22). We find the lower gains in FBFM we observed versus previous work (7, 10, 11) particularly perplexing given that: we used the identical training program, we ensured our participants had more than adequate dietary protein and energy intake (to result in an estimated positive energy balance), and we had participants that were of comparable training status based on FBFM and strength measures. However, we view the gains in FBFM we report as being more in line with typical gains (i.e., ~1.5kg) in lean mass for participants engaging in ~12 weeks of resistance training as reported in a recent meta-analysis from our laboratory (3).

Muscle thickness and CSA was assessed using ultrasound, which has been shown to be a reliable alternative to magnetic resonance imaging (15, 23). The Whey+HMB and Whey+Leu groups in our study demonstrated a ~5% increase in muscle thickness, which is in-line with a previous 12 wk RT study (15). In contrast to our results, Lowery et al (11) observed ~3 times greater increase in quadriceps muscle thickness of (7.8 ± 0.4 mm) in participants supplemented 3 g HMB-FA+ 400 mg ATP relative to a placebo group (2.4 ± 0.7 mm). The only other investigation to report such dramatic increases in muscle thickness was from the same study in a group receiving HMB-FA alone (10).

There is no real consensus on the ability of HMB to augment RT-induced muscle strength. Some trials report no effect (8, 24, 25), a trivial effect (26, 27) or considerable effect (7, 10, 11) of HMB to augment muscle mass and strength with RT. Previous work demonstrated significantly greater increases in total strength (3 times) for participants supplemented with HMB-FA (77 ± 18 kg) (10) and HMB+ATP (96 ± 8 kg) (11) compared to placebo (25 ± 22 kg). However, using the same training program as used previously (10, 11), we observed similar increases in total strength for Whey+HMB (70 ± 21 kg) and Whey+Leu (80 ± 40 kg). Following overreaching, both Whey+HMB and Whey+Leu experienced similar decrements in total strength (-0.5 ± 3 % and -2 ± 5 %) and subsequent recovery (5 ± 2 % and 6 ± 5 %). In addition, following overreaching, the Whey+HMB group experienced decrements in peak power that were, surprisingly, not observed in the Whey+Leu group indicating a superior ability of Whey+Leu to protect against declines in performance when overreaching. Again, our results are in sharp contrast with previous investigations (7, 10, 11) in which HMB markedly attenuated the declines in muscle strength and power following overreaching (10, 11). Our data indicate that HMB and Leu have a similar ability to augment RT-induced strength gains, attenuate decrements following overreaching, and facilitate recovery during a taper.

We report muscle hypertrophy and strength data that differ from recent investigations that utilized the same RT program (7, 10, 11). Nonetheless, our findings are analogous to preliminary investigations of HMB-Ca supplementation that did not observe a superior effect on hypertrophy and strength (8, 9). Nissen et al (9) examined HMB-Ca at varying doses 0 g, 1.5 g, 3 g in untrained participants. The authors suggested a dose-response effect on fat-free mass to increasing doses of HMB-Ca (9). Critically, there were no statistically significant increases in fat-free mass following HMB supplementation (9). In a subsequent investigation [Study 2 from (9)], trained participants were supplemented with 3 g HMB-Ca plus a nutrient powder (75 g protein) during 7 weeks of RT. The HMB+nutrient powder group increased fat-free mass on days 14-39 compared to controls (9). Nonetheless, the increase in fat-free mass was not significantly different between groups following training (9). In a separate study, 3 g of HMB-Ca was included in a protein supplement (75 g) given to trained participants, no difference was observed between HMB-Ca or protein groups (8). In spite of these results, the work of Nissen et al (9) is frequently, and incorrectly, cited to illustrate the anabolic effects of HMB (9). An independent effect of HMB on muscle mass accretion, due to the inclusion of protein in the same supplement, is impossible to elucidate from this trial (9). Critics attribute the discrepancies between these trials (8, 9) to the short supplementation protocol (28 days) and propose a longer period is required to elicit the anabolic effects of HMB (10, 11, 28). Nonetheless, we employed a highly effective training program used previously (7, 10, 11). Our data are in broad agreement with the conclusions of Kreider et al (8) who showed that a protein supplement with HMB does not enhance lean mass, strength or power compared to a protein supplement alone.

The training program used in the present and previous investigations (10, 11) is stated to induce muscle damage and a favourable hormone milieu upon which HMB would be maximally effective (7, 10, 11). The program, adapted from Kraemer et al (7), was designed to increase anabolic hormones by first performing multi-joint, compound lifts, followed by accessory lifts targeting smaller muscle groups. In fact, a single bout of training adapted from this program has been demonstrated to increase systemic hormones (29). Nonetheless, we and others (14, 30, 31) have showed that there is no anabolic effect that arises due to the acute, RT-induced rise in systemic hormones.

In the present investigation, we assessed various hormone concentrations at multiple time points and report no difference in hormones between groups at any phase. In addition, we assessed creatine kinase (CK), a frequently measured, but poor indicator of skeletal muscle damage (32, 33). A recent meta-analysis observed that HMB supplementation was effective in reducing serum levels of CK in studies ≥ 6 weeks (33). However, there is extensive debate as to the validity of CK, as serum levels are subject to substantial variation (32). The appearance of CK may reflect a disturbance to energy control processes and does not independently indicate structural damage to muscle cells (32). Following overreaching, CK (109 % and 72 %) and cortisol (47 % and 47 %) increased similarly in the Whey+HMB and Whey+Leu groups, respectively. The elevations in CK and cortisol recovered similarly during the 2-week taper. Our data support other investigations that have failed to observe any beneficial effect of HMB on CK release or cortisol (24, 34-37).

The principle strengths of the present study stem from the practical applications of our findings. We supplemented participants with whey protein because post-exercise consumption of a high-quality protein, such as whey, is standard nutritional practice (38, 39), and augments hypertrophy (3). We propose that persons undertaking RT to gain strength and muscle mass would not forgo the notable benefits of high-quality protein supplementation (3) and take only an isolated compound such as HMB, alone. Rather, we propose that most resistance exercisers would augment their nutritional program to include a supplement such as HMB in addition to a high-quality protein, especially given the ostensibly substantial anabolic advantage HMB has been shown to provide (7, 10, 11). We used multiple methods to assess changes muscle: FBFM by DXA, muscle thickness, muscle CSA, and muscle fiber CSA. There are, however, some limitations that are important to acknowledge. In the present analysis, we did not employ a control group. Although the mass and strength in the Whey+HMB and Whey+Leu groups are consistent with previous reports (3), we acknowledge that the inclusion of a non-supplemented control group would have improved the robustness of our findings. Secondly, we recognize that our supplementation protocol differs slightly from what is recommended for HMB: 1 g thrice daily as 30 minutes before exercise (8, 28). Finally, in contrast to previous investigations in which HMB-FA was used (10, 11), we utilized HMB in its calcium form (HMB-Ca), as this was the form associated with the greatest gains in muscle mass of ~9 kg versus only ~4 kg in the placebo group (7). However, we propose that the form of the HMB likely matters very little since recent work from Wilkinson et al (40) suggests that despite slightly differing bioavailability (41), HMB-Ca and HMB-FA are equivalent in their stimulation of MPS. And critically, HMB as a metabolite of leucine stimulates MPS, and inhibits muscle protein breakdown, through many of the same canonical signaling pathways as leucine (5, 40). Importantly, this is the first investigation to compare HMB to its parent metabolite during RT in young men and not merely a non-protein (usually carbohydrate) placebo. Indeed, our intention was to mimic the nutritional practices of athletes and recreational exercisers who frequently supplement with high-quality protein (38). We propose that our data is of practical relevance to athletes and recreational exercisers who hope to maximize their RT-induced gains through good nutritional and supplementation practices. In conclusion, our results show that there is no benefit of HMB when added to whey compared to whey protein with leucine.

**ACKNOWLEDGMENTS**

The authors declare that the results of the study are presented clearly, honestly and without fabrication, falsification or inappropriate data manipulation. The results of the present investigation do not constitute endorsement by ACSM. SMP declares that he has received competitive grant funding, honoraria, and travel expenses from the US National Dairy Council. No other authors declare any conflict of interest. This work was supported by internal McMaster University grant funds. EAN has received a grant from the Brazilian National Council for Scientific and Technological Development (CNPq) (Science without borders program) and is a CNPq Research Productivity Fellow. SMP receives grant funding from the National Science and Engineering Research Council of Canada and the Canadian Institutes for Health Research as well and the Canada Research Chairs program and acknowledges those sources of support during this investigation. The authors would like to thank Todd Prior (McMaster University) for technical assistance during the laboratory analyses and our participants for their participation and hard work.

**REFERENCES**

1. Phillips SM, Winett RA. Uncomplicated resistance training and health-related outcomes: evidence for a public health mandate. Curr Sports Med Rep. 2010;9(4):208-13. doi: 10.1249/JSR.0b013e3181e7da73. PubMed PMID: 20622538; PubMed Central PMCID: PMCPMC4086449.

2. American College of Sports Medicine. ACSM's guidelines for exercise testing and prescription. Lippincott Williams and Wilkins, 2013.

3. Morton RW, Murphy KT, McKellar SR, Schoenfeld BJ, Henselmans M, Helms E, et al. A systematic review, meta-analysis and meta-regression of the effect of protein supplementation on resistance training-induced gains in muscle mass and strength in healthy adults. Br J Sports Med. 2018;52(6): 376-384. Epub 2017/07/13. doi: 10.1136/bjsports-2017-097608. PubMed PMID: 28698222.

4. Phillips SM. The impact of protein quality on the promotion of resistance exercise-induced changes in muscle mass. Nutr Metab (Lond). 2016;13:64. Epub 2016/10/07. doi: 10.1186/s12986-016-0124-8. PubMed PMID: 27708684; PubMed Central PMCID: PMCPMC5041535.

5. Wilkinson DJ, Hossain T, Hill DS, Phillips BE, Crossland H, Williams J, et al. Effects of leucine and its metabolite beta-hydroxy-beta-methylbutyrate on human skeletal muscle protein metabolism. J Physiol. 2013;591(11):2911-23. doi: 10.1113/jphysiol.2013.253203. PubMed PMID: 23551944; PubMed Central PMCID: PMCPMC3690694.

6. Drummond MJ, Rasmussen BB. Leucine-enriched nutrients and the regulation of mammalian target of rapamycin signalling and human skeletal muscle protein synthesis. Curr Opin Clin Nutr Metab Care. 2008;11(3):222-6. doi: 10.1097/MCO.0b013e3282fa17fb. PubMed PMID: 18403916; PubMed Central PMCID: PMCPMC5096790.

7. Kraemer WJ, Hatfield DL, Volek JS, Fragala MS, Vingren JL, Anderson JM, et al. Effects of Amino Acids Supplement on Physiological Adaptations to Resistance Training. Med Sci Sport Exer. 2009;41(5):1111-21. doi: 10.1249/MSS.0b013e318194cc75. PubMed PMID: WOS:000265324500018.

8. Kreider RB, Ferreira M, Wilson M, Almada AL. Effects of calcium beta-hydroxy-beta-methylbutyrate (HMB) supplementation during resistance-training on markers of catabolism, body composition and strength. Int J Sports Med. 1999;20(8):503-9. doi: 10.1055/s-1999-8835. PubMed PMID: 10606212.

9. Nissen S, Sharp R, Ray M, Rathmacher JA, Rice D, Fuller JC, Jr., et al. Effect of leucine metabolite beta-hydroxy-beta-methylbutyrate on muscle metabolism during resistance-exercise training. J Appl Physiol (1985). 1996;81(5):2095-104. doi: 10.1152/jappl.1996.81.5.2095. PubMed PMID: 8941534.

10. Wilson JM, Lowery RP, Joy JM, Andersen JC, Wilson SM, Stout JR, et al. The effects of 12 weeks of beta-hydroxy-beta-methylbutyrate free acid supplementation on muscle mass, strength, and power in resistance-trained individuals: a randomized, double-blind, placebo-controlled study. Eur J Appl Physiol. 2014;114(6):1217-27.

11. Lowery RP, Joy JM, Rathmacher JA, Baier SM, Fuller JC, Jr., Shelley MC, 2nd, et al. Interaction of Beta-Hydroxy-Beta-Methylbutyrate Free Acid and Adenosine Triphosphate on Muscle Mass, Strength, and Power in Resistance Trained Individuals. J Strength Cond Res. 2016;30(7):1843-54. doi: 10.1519/JSC.0000000000000482. PubMed PMID: 24714541.

12. Phillips SM, Aragon AA, Arciero PJ, Arent SM, Close GL, Hamilton DL, et al. Changes in body composition and performance with supplemental HMB-FA+ATP. J Strength Cond Res.107;31(5): e71-e72. Epub 2017/03/17. doi: 10.1519/jsc.0000000000001760. PubMed PMID: 28301440.

13. Kraemer WJ, Hatfield DL, Comstock BA, Fragala MS, Davitt PM, Cortis C, et al. Influence of HMB supplementation and resistance training on cytokine responses to resistance exercise. J Am Coll Nutr. 2014;33(4):247-55. doi: 10.1080/07315724.2014.911669. PubMed PMID: 25140763.

14. Morton RW, Oikawa SY, Wavell CG, Mazara N, McGlory C, Quadrilatero J, et al. Neither load nor systemic hormones determine resistance training-mediated hypertrophy or strength gains in resistance-trained young men. J Appl Physiol (1985). 2016;121(1):129-38. doi: 10.1152/japplphysiol.00154.2016. PubMed PMID: 27174923; PubMed Central PMCID: PMCPMC4967245.

15. Franchi MV, Longo S, Mallinson J, Quinlan JI, Taylor T, Greenhaff PL, et al. Muscle thickness correlates to muscle cross-sectional area in the assessment of strength training-induced hypertrophy. Scand J Med Sci Sports. 2017;28(3):846-853. doi: 10.1111/sms.12961. PubMed PMID: 28805932.

16. Ruas CV, Brown LE, Lima CD, Costa PB, Pinto RS. Effect of three different muscle action training protocols on knee strength ratios and performance. J Strength Cond Res. 2018;32(8):2154-2165. doi: 10.1519/JSC.0000000000002134. PubMed PMID: 28704309.

17. Lixandrao ME, Ugrinowitsch C, Bottaro M, Chacon-Mikahil MP, Cavaglieri CR, Min LL, et al. Vastus lateralis muscle cross-sectional area ultrasonography validity for image fitting in humans. J Strength Cond Res. 2014;28(11):3293-7. doi: 10.1519/JSC.0000000000000532. PubMed PMID: 24845210.

18. Shenouda N, Proudfoot NA, Currie KD, Timmons BW, MacDonald MJ. Automated ultrasound edge-tracking software comparable to established semi-automated reference software for carotid intima-media thickness analysis. Clin Physiol Funct Imaging. 2018;38(3):396-401. doi: 10.1111/cpf.12428. PubMed PMID: 28444941.

19. Burgomaster KA, Hughes SC, Heigenhauser GJ, Bradwell SN, Gibala MJ. Six sessions of sprint interval training increases muscle oxidative potential and cycle endurance capacity in humans. J Appl Physiol (1985). 2005;98(6):1985-90. doi: 10.1152/japplphysiol.01095.2004. PubMed PMID: 15705728.

20. Bloemberg D, Quadrilatero J. Rapid determination of myosin heavy chain expression in rat, mouse, and human skeletal muscle using multicolor immunofluorescence analysis. PLoS One. 2012;7(4):e35273. doi: 10.1371/journal.pone.0035273. PubMed PMID: 22530000; PubMed Central PMCID: PMCPMC3329435.

21. McGuigan MR, Kraemer WJ, Deschenes MR, Gordon SE, Kitaura T, Scheett TP, et al. Statistical analysis of fiber area in human skeletal muscle. Can J Appl Physiol. 2002;27(4):415-22.

22. Gentles JA, Phillips SM. Discrepancies in publications related to HMB-FA and ATP supplementation. Nutr Metab (Lond). 2017;14:42. Epub 2017/07/07. doi: 10.1186/s12986-017-0201-7. PubMed PMID: 28680453; PubMed Central PMCID: PMCPMC5496306.

23. Reeves ND, Maganaris CN, Narici MV. Ultrasonographic assessment of human skeletal muscle size. Eur J Appl Physiol. 2004;91(1):116-8. doi: 10.1007/s00421-003-0961-9. PubMed PMID: 14639480.

24. Slater G, Jenkins D, Logan P, Lee H, Vukovich M, Rathmacher JA, et al. Beta-hydroxy-beta-methylbutyrate (HMB) supplementation does not affect changes in strength or body composition during resistance training in trained men. Int J Sport Nutr Exerc Metab. 2001;11(3):384-96. PubMed PMID: 11599506.

25. Paddon-Jones D, Keech A, Jenkins D. Short-term beta-hydroxy-beta-methylbutyrate supplementation does not reduce symptoms of eccentric muscle damage. Int J Sport Nutr Exerc Metab. 2001;11(4):442-50. PubMed PMID: 11915779.

26. Thomson JS, Watson PE, Rowlands DS. Effects of nine weeks of beta-hydroxy-beta- methylbutyrate supplementation on strength and body composition in resistance trained men. J Strength Cond Res. 2009;23(3):827-35. doi: 10.1519/JSC.0b013e3181a00d47. PubMed PMID: 19387396.

27. Durkalec-Michalski K, Jeszka J. The Effect of beta-Hydroxy-beta-Methylbutyrate on Aerobic Capacity and Body Composition in Trained Athletes. J Strength Cond Res. 2016;30(9):2617-26. doi: 10.1519/JSC.0000000000001361. PubMed PMID: 26849784.

28. Wilson JM, Fitschen PJ, Campbell B, Wilson GJ, Zanchi N, Taylor L, et al. International Society of Sports Nutrition Position Stand: beta-hydroxy-beta-methylbutyrate (HMB). J Int Soc Sports Nutr. 2013;10(1):6. doi: 10.1186/1550-2783-10-6. PubMed PMID: 23374455; PubMed Central PMCID: PMCPMC3568064.

29. Wilson JM, Lowery RP, Joy JM, Walters JA, Baier SM, Fuller JC, Jr., et al. beta-Hydroxy-beta-methylbutyrate free acid reduces markers of exercise-induced muscle damage and improves recovery in resistance-trained men. Br J Nutr. 2013;110(3):538-44.

30. West DW, Kujbida GW, Moore DR, Atherton P, Burd NA, Padzik JP, et al. Resistance exercise-induced increases in putative anabolic hormones do not enhance muscle protein synthesis or intracellular signalling in young men. J Physiol. 2009;587(Pt 21):5239-47. doi: 10.1113/jphysiol.2009.177220. PubMed PMID: 19736298; PubMed Central PMCID: PMCPMC2790261.

31. West DW, Cotie LM, Mitchell CJ, Churchward-Venne TA, MacDonald MJ, Phillips SM. Resistance exercise order does not determine postexercise delivery of testosterone, growth hormone, and IGF-1 to skeletal muscle. Appl Physiol Nutr Metab. 2013;38(2):220-6. doi: 10.1139/apnm-2012-0397. PubMed PMID: 23438236.

32. Baird MF, Graham SM, Baker JS, Bickerstaff GF. Creatine-kinase- and exercise-related muscle damage implications for muscle performance and recovery. J Nutr Metab. 2012;2012:960363. doi: 10.1155/2012/960363. PubMed PMID: 22288008; PubMed Central PMCID: PMCPMC3263635.

33. Rahimi MH, Mohammadi H, Eshaghi H, Askari G, Miraghajani M. The Effects of Beta-Hydroxy-Beta-Methylbutyrate Supplementation on Recovery Following Exercise-Induced Muscle Damage: A Systematic Review and Meta-Analysis. J Am Coll Nutr. 2018:1-10. doi: 10.1080/07315724.2018.1451789. PubMed PMID: 29676656.

34. Knitter AE, Panton L, Rathmacher JA, Petersen A, Sharp R. Effects of beta-hydroxy-beta-methylbutyrate on muscle damage after a prolonged run. J Appl Physiol (1985). 2000;89(4):1340-4. doi: 10.1152/jappl.2000.89.4.1340. PubMed PMID: 11007567.

35. Crowe MJ, O'Connor DM, Lukins JE. The effects of beta-hydroxy-beta-methylbutyrate (HMB) and HMB/creatine supplementation on indices of health in highly trained athletes. Int J Sport Nutr Exerc Metab. 2003;13(2):184-97. PubMed PMID: 12945829.

36. Durkalec-Michalski K, Jeszka J. The efficacy of a beta-hydroxy-beta-methylbutyrate supplementation on physical capacity, body composition and biochemical markers in elite rowers: a randomised, double-blind, placebo-controlled crossover study. J Int Soc Sports Nutr. 2015;12:31. doi: 10.1186/s12970-015-0092-9. PubMed PMID: 26225130; PubMed Central PMCID: PMCPMC4518594.

37. Durkalec-Michalski K, Jeszka J, Podgorski T. The Effect of a 12-Week Beta-hydroxy-beta-methylbutyrate (HMB) Supplementation on Highly-Trained Combat Sports Athletes: A Randomised, Double-Blind, Placebo-Controlled Crossover Study. Nutrients. 2017;9(7). doi: 10.3390/nu9070753. PubMed PMID: 28708126; PubMed Central PMCID: PMCPMC5537867.

38. Kristiansen M, Levy-Milne R, Barr S, Flint A. Dietary supplement use by varsity athletes at a Canadian university. Int J Sport Nutr Exerc Metab. 2005;15(2):195-210. PubMed PMID: 16089277.

39. Froiland K, Koszewski W, Hingst J, Kopecky L. Nutritional supplement use among college athletes and their sources of information. Int J Sport Nutr Exerc Metab. 2004;14(1):104-20. PubMed PMID: 15129934.

40. Wilkinson DJ, Hossain T, Limb MC, Phillips BE, Lund J, Williams JP, et al. Impact of the calcium form of beta-hydroxy-beta-methylbutyrate upon human skeletal muscle protein metabolism. Clin Nutr. 2017. doi: 10.1016/j.clnu.2017.09.024. PubMed PMID: 29097038.

41. Fuller JC, Jr., Sharp RL, Angus HF, Baier SM, Rathmacher JA. Free acid gel form of beta-hydroxy-beta-methylbutyrate (HMB) improves HMB clearance from plasma in human subjects compared with the calcium HMB salt. Br J Nutr. 2011;105(3):367-72.

**Figure Legends**

Figure 1. A) Absolute values pre- and post training and B) Change in fat-and bone free mass (FBFM) for Whey+HMB (n=13, open box) and Whey+Leu (n=13, grey box) following 12 weeks of resistance training. Values are presented as median (lines) with the interquartile range (boxes) ± minimum and maximum values (whiskers) and where + indicates the mean. *Significantly different (p<0.05) from pre-training.

Figure 2. A) Absolute values pre- and post-training and B) Change in *vastus lateralis* muscle thickness for Whey+HMB (n=13, open box) and Whey+Leu (n=12, grey box) following 12 weeks of undulating periodized resistance training. C) Absolute change and D) Delta change in *vastus lateralis* cross-sectional area for Whey+HMB and Whey+Leu. Values are presented as median (lines) with the interquartile range (boxes) ± minimum and maximum values (whiskers) and where + indicates the mean. *Significantly different (p<0.05) from pre-training.

**Supplementary Digital Content**

Supplementary Digital content 1. pdf

Figure 1. Group allocation

Supplementary Digital content 2. pdf

Figure 2. Schematic representation of study design
