# Supplementary figures and images for: Wrist-worn Accelerometry for Runners: Objective Quantification of Training Load
Source: Med Sci Sports Exerc. 2018 Jul 30;50(11):2277–84. doi: 10.1249/MSS.0000000000001704 (PMC6195805; doi:10.1249/MSS.0000000000001704)

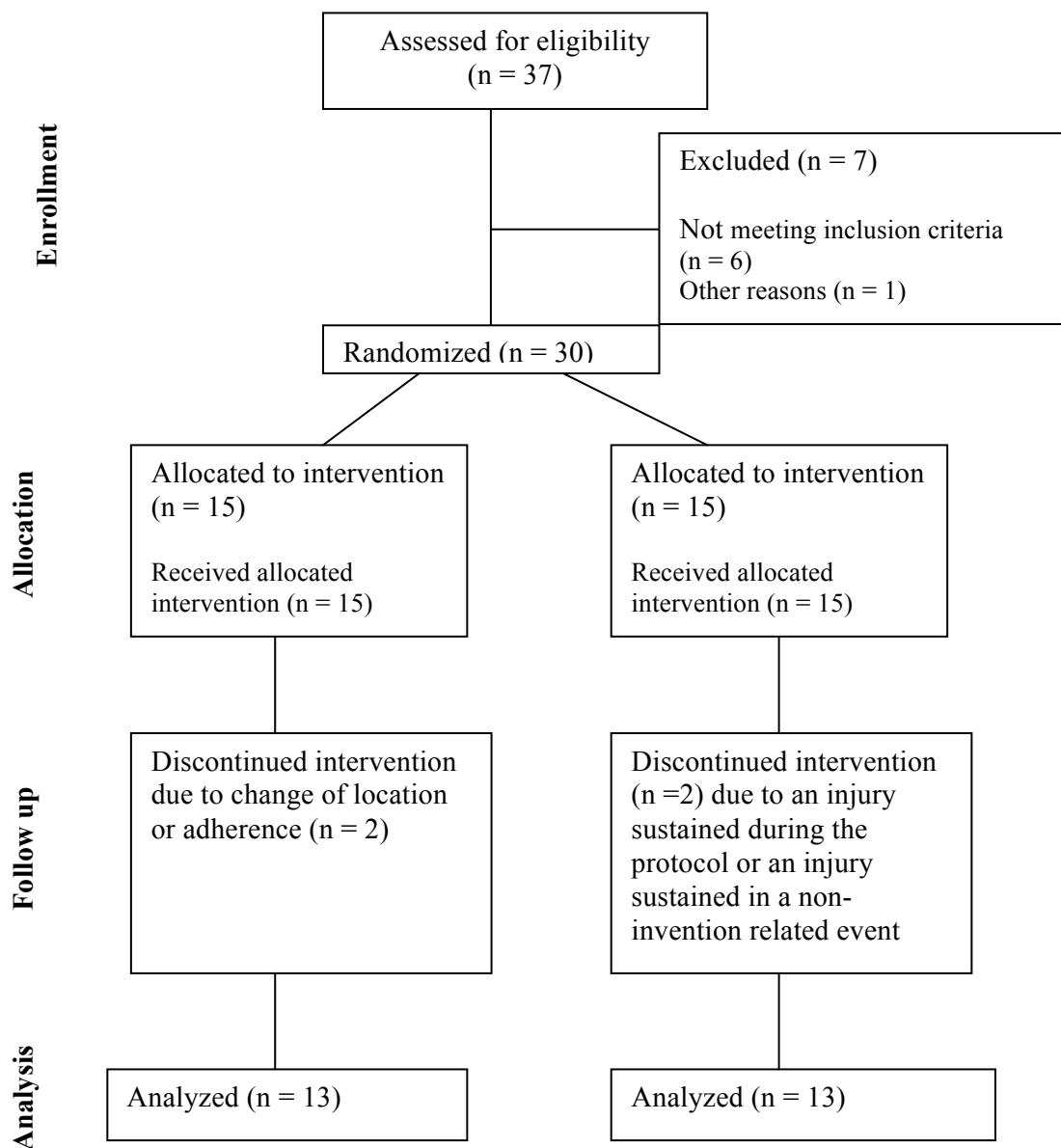

SDC Figure 1. Group Allocation

Supplement: SUPPLEMENTARY MATERIAL [file mss-50-2277-s002.pdf]

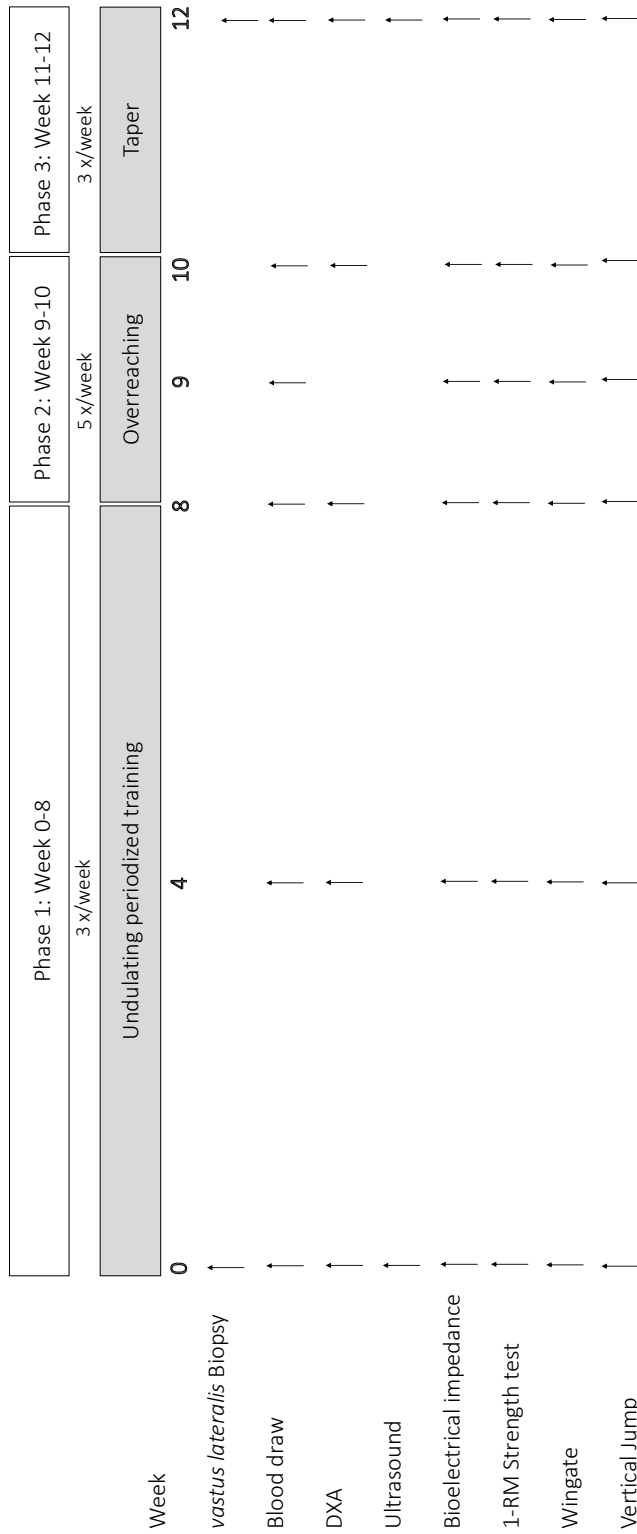

SDC Figure 2. Schematic representation of Study design

Supplement: SUPPLEMENTARY MATERIAL [file mss-50-2277-s003.pdf]
